# Identification of Trans-4-Hydroxy-L-Proline as a Compatible Solute and Its Biosynthesis and Molecular Characterization in *Halobacillus halophilus*

**DOI:** 10.3389/fmicb.2017.02054

**Published:** 2017-10-20

**Authors:** Kyung Hyun Kim, Baolei Jia, Che Ok Jeon

**Affiliations:** Department of Life Science, Chung-Ang University, Seoul, South Korea

**Keywords:** compatible solute, trans-4-hydroxy-L-proline, proline 4-hydroxylase, transcriptional regulation, *Halobacillus halophilus*

## Abstract

*Halobacillus halophilus*, a moderately halophilic bacterium, accumulates a variety of compatible solutes including glycine betaine, glutamate, glutamine, proline, and ectoine to cope with osmotic stress. Non-targeted analysis of intracellular organic compounds using ^1^H-NMR showed that a large amount of trans-4-hydroxy-L-proline (Hyp), which has not been reported as a compatible solute in *H. halophilus*, was accumulated in response to high NaCl salinity, suggesting that Hyp may be an important compatible solute in *H. halophilus*. Candidate genes encoding proline 4-hydroxylase (PH-4), which hydroxylates L-proline to generate Hyp, were retrieved from the genome of *H. halophilus* through domain searches based on the sequences of known PH-4 proteins. A gene, HBHAL_RS11735, which was annotated as a multidrug DMT transporter permease in GenBank, was identified as the PH-4 gene through protein expression analysis in *Escherichia coli*. The PH-4 gene constituted a transcriptional unit with a promoter and a rho-independent terminator, and it was distantly located from the proline biosynthetic gene cluster (*pro* operon). Transcriptional analysis showed that PH-4 gene expression was NaCl concentration-dependent, and was specifically induced by chloride anion, similar to the *pro* operon. Accumulation of intracellular Hyp was also observed in other bacteria, suggesting that Hyp may be a widespread compatible solute in halophilic and halotolerant bacteria.

## Introduction

Halotolerant or halophilic microorganisms have become well adapted to high salinity environments such as, soda lakes, coastal lagoons, and solar salterns by evolving various strategies to cope with the osmotic stress associated with high salt conditions (Ventosa et al., 1998; Oren, 2015). As one such strategy, these microbes actively adjust their cytoplasmic osmolarity by accumulating osmolytes such as, salts or compatible solutes. Generally, halophilic *Archaea* achieve osmotic balance by increasing intracellular salt (e.g., KCl) concentrations in response to high salinity, whereas halophilic and halotolerant bacteria typically accumulate compatible solutes within their cytoplasm to maintain osmotic balance (Mongodin et al., 2005; Shivanand and Mugeraya, 2011; Hänelt and Müller, 2013). However, a hybrid strategy, in which salts and compatible solutes are accumulated to cope with increasing salinity, has also been adopted by many moderately halophilic bacteria, such as, *Halobacillus halophilus*, that grow optimally between 0.5 and 2.0 M NaCl (Saum et al., 2013).

Compatible solutes are intracellular organic compounds that halophilic and halotolerant bacteria accumulate to protect themselves from osmotic stress caused by high NaCl concentrations (Galinski and Trüper, 1994; Roberts, 2005). Most common compatible solutes used to cope with high salinity are polyhydroxylated organic compounds that are polar, highly water-soluble, and carry no net charge at physiological pH, such as, glutamate, glutamine, glycine betaine, trehalose, proline, ectoine, β-hydroxyectoine, and mannitol. These solutes have been reported in a variety of halophilic and halotolerant bacteria, including *H. halophilus, Bacillus subtilis, Acinetobacter baylyi*, and *Vibrio parahaemolyticus* (Kempf and Bremer, 1998; Hänelt and Müller, 2013; Ongagna-Yhombi and Boyd, 2013; Sand et al., 2013; Zaprasis et al., 2015; Sévin et al., 2016). The inhibitory effects of these solutes on vital cellular functions are small even when present in the cytoplasm at high concentrations.

*H. halophilus* DSM 2266, originally described as *Sporosarcina halophila*, is a rod-shaped, endospore-forming, gram-positive bacterium that was isolated from a salt marsh on the North Sea coast of Germany (Claus et al., 1983; Spring et al., 1996), and it has been extensively studied as a well-known model microorganism for osmoadaptation. Under high salinity conditions, glutamate, glutamine, proline, ectoine, and glycine betaine are accumulated as major compatible solutes in *H. halophilus*, and their biosynthetic pathways and biochemical properties have been well studied (Hänelt and Müller, 2013). The growth of *H. halophilus* is strictly dependent on NaCl concentration, specifically chloride anion, which regulates the physiological responses to osmotic stress, such as, stimulation of flagellar synthesis, motility (Roeßler et al., 2000), and endospore germination (Dohrmann and Müller, 1999), in high salt environments. In addition, chloride anion modulates the activity of the glycine betaine transporter (Roeßler and Müller, 2001) and the biosynthesis of various compatible solutes, including glycine betaine, glutamate, glutamine, proline, and ectoine (Hänelt and Müller, 2013).

Although trans-4-hydroxy-L-proline (Hyp) has been reported to accumulate in *Thermococcus litoralis* and *Brevibacterium* sp. JCM 6894 under high salt conditions (Nagata et al., 1996; Lamosa et al., 1998), it has not been considered to be an important compatible solute for coping with high salinity in *H. halophilus*. Moreover, the biosynthesis and regulation of Hyp in response to high NaCl salinity has not yet explored even in other bacteria. In this study, we observed that *H. halophilus* accumulated a large amount of Hyp using ^1^H-NMR spectroscopy and we identified and characterized a proline 4-hydroxylase (PH-4) gene in the genome of *H. halophilus* that is essential for the biosynthesis of Hyp from proline. We also investigated the transcriptional regulation of the PH-4 gene in response to high salinity. In addition, Hyp accumulation under high NaCl conditions in other halotolerant and halophilic bacteria was investigated to evaluate the possibility of Hyp as a universal compatible solute.

## Materials and methods

### Cultivation of *H. halophilus* DSM 2266

*H. halophilus* DSM 2266^T^ was routinely grown in marine broth 2216 (MB; BD, USA) supplemented with NaCl to a final concentration of 10% (w/v) at 30°C in a rotary shaker with 220 rpm. To analyze the compatible solutes and the salt-dependence of trans-4-hydroxy- L-proline (Hyp) biosynthetic gene transcriptions, the concentrations of NaCl (or other salts) added to the MB was varied as described in each assay.

### Identification of new compatible solutes in *H. halophilus* using ^1^H-NMR

To search for compatible solutes in *H. halophilus* that respond to high salinity, cells were grown in MB containing 0.4, 1.0, or 2.5 M NaCl and harvested during mid-exponential phase (OD_600_ = 0.6–0.8) by centrifugation. The harvested cells were lyophilized and their intracellular osmolytes were extracted using a modification of method reported by Bligh and Dyer (Kunte et al., 1993; Saum et al., 2006). Briefly, 200 mg of the lyophilized cells was resuspended in 570 μl of extraction solution [methanol: chloroform: water, 10:5:4 (vol/vol/vol)] and the mixture was homogenized for 2 min with a Fast-Prep instrument (MP Biomedicals, USA). Then, 170 μl of chloroform and 170 μl of water were added, and the resulting mixture was homogenized for 2 min. Following phase separation of the mixture by centrifugation aqueous top layer was removed and dried.

Total intracellular organic compounds, including compatible solutes, in the dried residues were analyzed using ^1^H-NMR spectroscopy, according to a previously described procedure (Lee et al., 2012; Sand et al., 2013). Briefly, the dried residues were dissolved in 600 μl of 99.9% D_2_O (Sigma-Aldrich, USA) containing 5 mM sodium 2,2-dimethyl-2-silapentane-5-sulfonate (DSS; Sigma-Aldrich, USA), and then centrifuged at 12,000 × g for 5 min. The supernatants were transferred to 5-mm NMR tubes, and their ^1^H-NMR spectra were acquired with a Varian Inova 600-MHz NMR spectrometer (Varian, USA). Identification and quantification of intracellular organic compounds from the ^1^H-NMR spectra were performed using Chenomx NMR suite (ver. 6.1; Chenomx, Canada).

### Determination of compatible solutes in *H. halophilus* by HPLC

*H. halophilus* cells were grown in MB containing different NaCl concentrations (0.4, 1.0, 1.5, 2.0, 2.5, or 3.0 M) and harvested during mid-exponential phase (OD_600_ = 0.6–0.8). Compatible solutes from the harvested cells were extracted as described above. Dried residues containing compatible solutes were resuspended in 500 μl of distilled water (DW), and the resulting solutions were filtered through an Ultracel-3K filter (Millipore, USA) to remove undissolved high-molecular-weight compounds including residual proteins. The resulting filtrates were derivatized with 9-fluorenylmethoxy carbonyl (FMOC), according to a previously described method (Kunte et al., 1993). Briefly, 150 μl of FMOC reagent (1.5 mM in acetone) and 150 μl of sample or standard (glutamate, glutamine, proline, and Hyp) were added to 150 μl of sodium borate buffer (0.5 M, pH 7.7) containing 20 μM norvaline as an internal standard, and then vigorously mixed. Then, 200 μl of amantadine HCl reagent (12 mM in borate buffer) was added to the mixture to remove excess FMOC.

FMOC-derivatized compatible solutes were analyzed using an HPLC system (Shimadzu, Japan) equipped with a reverse-phased Kromasil C_18_ column (250 × 4.6 mm; Akzo Nobel, Sweden) and a fluorescence detector (RF-10AXL; Shimadzu, Japan), according to the procedure described by Saum et al. (2006). Total proteins from cells grown in MB containing different NaCl concentrations were assayed according to a previously described procedure (Saum et al., 2006), and the concentrations of the compatible solutes were calculated based on the total protein content.

### Identification of candidate genes encoding proline 4-hydroxylase in *H. halophilus*

We searched the whole genome of *H. halophilus* (GenBank acc. no. NC_017668.1–NC_017670.1) for candidate PH-4-encoding genes catalyzing the conversion of proline to Hyp using the α-ketoglutarate- and Fe (II)-dependent oxygenase (2OG-Fe[II] Oxy) domain sequences of the PH-4 proteins from *Dactylosporangium* sp., *Pseudomonas stutzeri*, and *Bordetella bronchiseptica* RB5 by NCBI BLASTP and the DELTA-BLAST algorithm. The presence of 2OG-Fe[II] Oxy domain sequences in the candidate PH-4 genes was confirmed with NCBI Conserved Domain BLAST (Marchler-Bauer et al., 2015) and the Pfam protein families database (Finn et al., 2014). A multiple amino acid sequence alignment of the PH-4 candidates and PH-4 homologs in GenBank was performed using ClustalW (Thompson et al., 2002), and the alignment was visualized using Circoletto with an E-value ≤ 1 e−5 (Darzentas, 2010). An unrooted phylogenetic tree was also constructed using MEGA6 based on the maximum likelihood algorithm (Tamura et al., 2013), and the tree topology was statistically evaluated through bootstrap analysis using 1,000 replicates.

### Overexpression and biochemical analysis of candidate PH-4 genes

Three candidate PH-4 genes in the genome of *H. halophilus* with the GenBank locus tags HBHAL_RS02420, HBHAL_RS11735, and HBHAL_RS15600 were cloned into the pET-28a expression vector (Novagen, USA) for overexpression. Briefly, the candidate genes were PCR amplified from *H. halophilus* genomic DNA with Pfu DNA polymerase (Solgent, Korea) and specific cloning primer sets (Supplementary Table 1). The PCR products and the pET-28a vector were digested with the appropriate restriction enzymes and ligated together. Then, the constructs were transformed into *Escherichia coli* BL21 (DE3) competent cells by electroporation, and the *E. coli* transformants were confirmed by PCR and DNA sequencing.

*Escherichia coli* BL21 (DE3) cells carrying the candidate PH-4 genes were cultured in 500 mL of LB broth containing kanamycin (30 μg/ml) at 37°C with shaking to an OD_600_ of ~0.7. The cultures were induced with 1 mM isopropyl-β-d-thiogalactopyranoside (IPTG) and incubated for 4 h. Then, cells were harvested by centrifugation (10,000 rpm for 10 min at 4°C). The overexpression of the candidate PH-4 genes in *E. coli* was confirmed by 12% SDS-PAGE. To measure PH-4 enzyme activity, the harvested cells were resuspended to a final cell density of ~0.1% (wet cell basis) in 50 mM 2-[*N*-morpholino] ethanesulfonic acid buffer (pH 6.5) containing 4 mM L-proline, 8 mM α-ketoglutarate, 2 mM FeSO_4_, and 4 mM l-ascorbic acid. The reaction mixtures were incubated at 30°C for 10 min and then inactivated by heat treatment at 100°C for 5 min. After centrifugation, the amount of Hyp produced by the cell supernatants was determined by HPLC as described above for the analysis of compatible solutes. One unit was defined as the amount of PH-4 that forms 1 nmol of Hyp per min at 30°C. *E. coli* BL21 (DE3) cells without the candidate genes and *H. halophilus* DSM 2266 were used as controls.

### Analysis of proline and Hyp biosynthetic gene clusters in *H. halophilus*

The proline and Hyp biosynthetic gene clusters in the genome of *H. halophilus* were identified using BLASTX and BLASTN and their transcriptional promoters and terminators were predicted using the web-based programs, BPROM and FindTerm, respectively, available at http://www.softberry.com/.

### Transcriptional analysis of the proline and Hyp biosynthetic genes in *H. halophilus*

*H. halophilus* DSM 2266 cells were cultivated in MB containing different NaCl concentrations (0.4, 1.0, 1.5, 2.0, 2.5, or 3.0 M) or salts (NaCl, NaBr, or NaNO_3_ added at 1 M to MB containing 0.4 M NaCl), harvested in early exponential phase (OD_600_ = 0.15–0.3), and stored at −80°C until analysis. Transcriptional expression of the proline and Hyp biosynthetic genes was analyzed in triplicate as described previously (Saum et al., 2006; Saum and Müller, 2007), with some modifications. Total RNA was extracted from frozen cells using TRIzol® reagent (Life Technologies, USA) according to the manufacturer's instructions, and the extracted total RNA was treated with RNase-free DNase I (Qiagen, USA) to eliminate genomic DNA contamination. HBHAL_RS03960 [annotated as pyrroline-5-carboxylate reductase (*proH*)] and HBHAL_RS11735 (annotated as a multidrug DMT transporter permease) were targeted for transcriptional analysis of the proline and Hyp biosynthetic genes, respectively. The transcriptional expression of malate dehydrogenase (HBHAL_RS13485, *mdh*) was used for normalization to total RNA. Reverse transcriptase quantitative PCR (RT-qPCR) of the target genes was carried out using the iScript One-Step RT-PCR kit with SYBR Green (Bio-Rad, USA) and specific primer sets (Supplementary Table 1) in a C1000 Thermal Cycler (Bio-Rad, USA), as described previously (Lee et al., 2011). The copy numbers of the gene transcripts were analyzed by the 2(-Delta Delta C(T)) method (Livak and Schmittgen, 2001), and their relative expression levels in MB containing different NaCl concentrations and salts were calculated based on their expression levels in MB containing 0.4 M NaCl and 1.0 M NaCl, respectively.

### Analysis of intracellular proline and Hyp in various halotolerant and halophilic bacteria

The intracellular accumulation of proline and Hyp in various halotolerant and halophilic bacteria, including *B. subtilis* KACC 17796^T^, *Virgibacillus pantothenticus* KCTC 3860^T^, *Virgibacillus siamensis* JCM 15395^T^, *Lentibacillus juripiscarius* JCM 12147^T^, *L. salicampi* KACC 17232^T^, *Salimicrobium halophilum* JCM 12305^T^, *Maribacter stanieri* KACC 15388^T^, *B. bronchiseptica* KACC 11941^T^, *P. stutzeri* KACC 10290^T^, and *Halomonas elongata* KCTC 3541^T^, was investigated. The test strains were cultivated in MB containing optimum and stress (corresponding to 1.5–3 times the optimum NaCl concentrations) salinity levels. The concentrations of intracellular proline and Hyp in harvested cells were analyzed in triplicate using HPLC, as described above.

## Results

### Identification of new compatible solutes in *H. halophilus* using ^1^H-NMR

**Figure 1:**
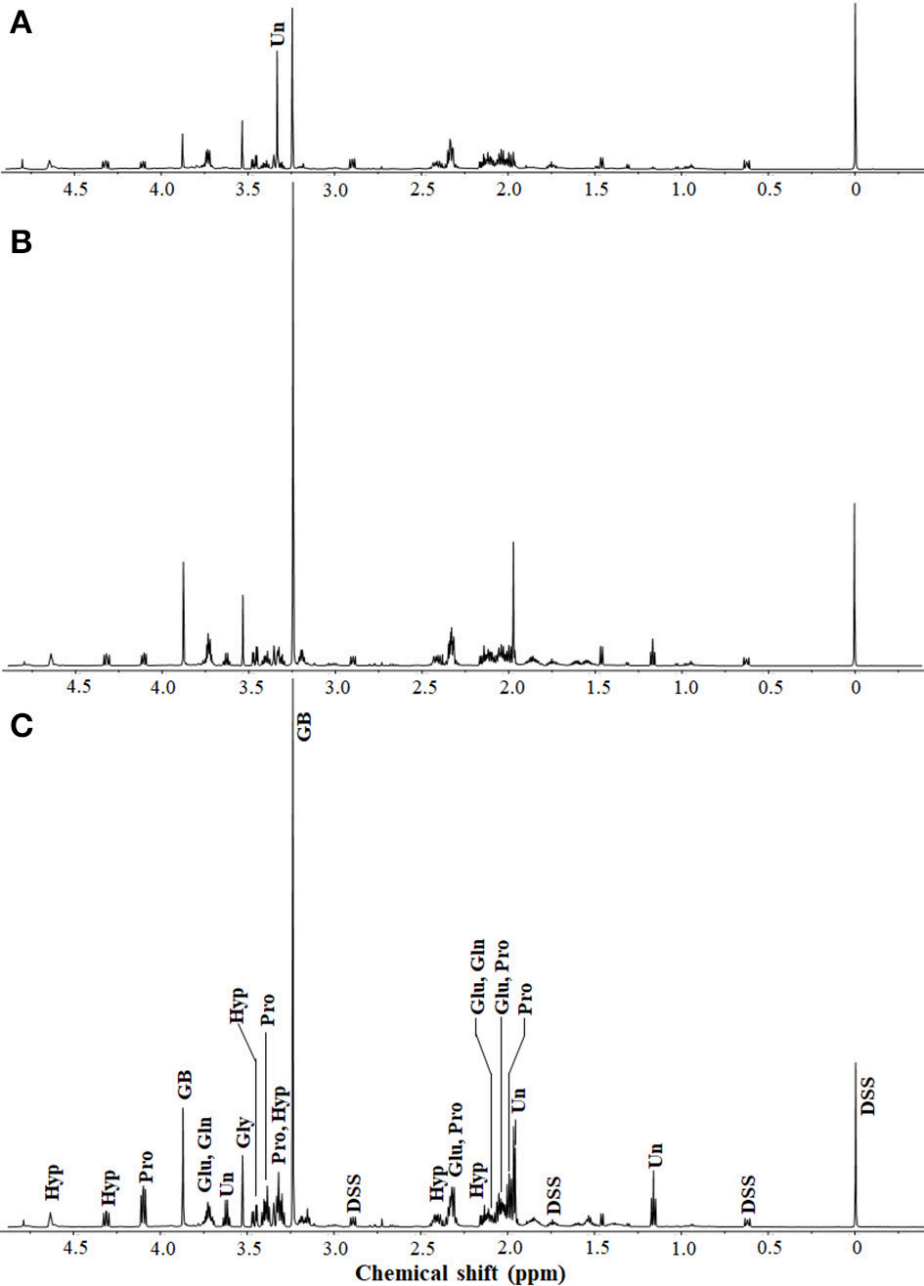
^1^H-NMR spectra of extracts from *H. halophilus* cells grown in marine broth containing 0.4 **(A)**, 1.0 **(B)**, and 2.5 M **(C)** NaCl. Sodium 2,2-dimethyl-2-silapentane-5-sulfonate (DSS; 5 mM) was used as the internal standard. Intracellular organic compounds from the ^1^H-NMR spectra were identified with the Chenomx NMR suite. ^1^H-NMR peaks corresponding to glutamate (Glu), glutamine (Gln), glycine betaine (GB), proline (Pro), glycine (Gly), and trans-4-hydroxy- L-proline (Hyp) are indicated. Unidentified intracellular organic compounds (Un) that responded to changes in NaCl concentrations are also shown.

To investigate all compatible solutes responding to high salinity in *H. halophilus*, the intracellular organic compounds from cells grown in MB containing different NaCl concentrations were analyzed using ^1^H-NMR spectroscopy. Glutamate, glutamine, glycine betaine, and proline, which are well known compatible solutes in *H. halophilus*, were identified as the major organic compounds responding to increasing NaCl concentrations (Burkhardt et al., 2009; Hänelt and Müller, 2013; Figure 1). The concentrations of glycine betaine, glutamine, and proline gradually increased as the NaCl concentration increased. However, although the concentration of glutamate was higher at 1.0 M NaCl than at 0.4 M NaCl, it decreased at 2.5 M NaCl (Supplementary Figure 1).

In addition to these known compatible solutes, the non-targeted ^1^H-NMR analysis identified other organic compounds that were also increased as the NaCl concentration increased. Glycine, represented as a singlet at 3.5 ppm, was detected as an intracellular organic compound responding to increased NaCl concentration, although the increase was not great. The ^1^H-NMR peak areas at 2.1, 2.4, 3.4, 4.3, and 4.7 ppm, which correspond to Hyp in the compound library of the Chenomx NMR suite, gradually increased as the NaCl concentration increased. At 2.5 M NaCl, the Hyp concentration was ~2.14-fold higher than that at 0.4 M NaCl (Supplementary Figure 1). In addition, the ^1^H-NMR analysis showed some other unidentified organic compounds responding to increasing NaCl concentrations. The ^1^H-NMR peak at 3.34 ppm was one of the major peaks at 0.4 M NaCl; however, it was not detected at 1.0 or 2.5 M NaCl. Additional ^1^H-NMR peaks, including those at 1.2, 1.97, 1.98, and 3.6 ppm, that were weak at 0.4 M NaCl were relatively strong at 1.0 and 2.5 M NaCl, suggesting the presence of other unknown putative compatible solutes in *H. halophilus*.

**Figure 2:**
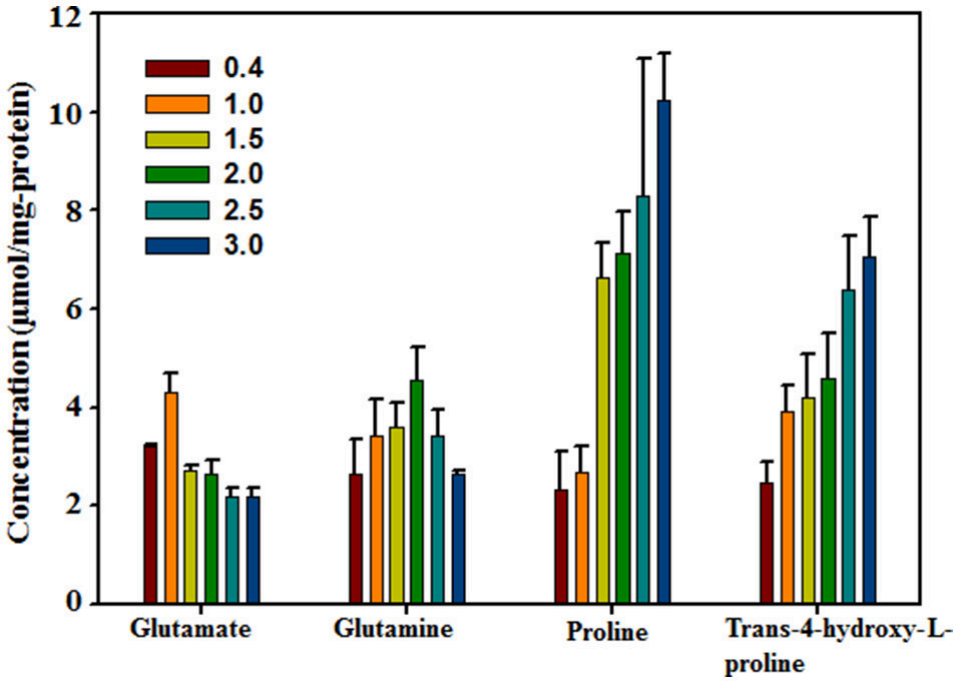
Concentrations of major intracellular organic compounds in *H. halophilus* grown in marine broth with different NaCl concentrations as quantified by HPLC.

To confirm Hyp as an important compatible solute that responds to high salinity, the intracellular Hyp contents of *H. halophilus* grown in MB containing different NaCl concentrations were analyzed using HPLC along with the known compatible solutes glutamate, glutamine, and proline (Figure 2). The NaCl concentration-dependent concentration profiles of these intracellular organic compounds were in relatively good accordance with the results shown in the above ^1^H-NMR analysis as well as those of a previous study (Saum and Müller, 2007). At lower NaCl concentrations, the concentrations of glutamate and glutamine increased as the NaCl concentration increased; however, at higher NaCl concentrations, their concentrations decreased slightly. In contrast, the intracellular concentrations of proline and Hyp steadily increased as the NaCl concentration increased, indicating that they were clearly NaCl-concentration dependent. The intracellular Hyp concentration increased ~2.85-fold from 0.4 M NaCl to 3.0 M NaCl (2.48 μmol/mg protein at 0.4 M NaCl and 7.07 μmol/mg protein at 3.0 M NaCl). This salinity-dependent increase in intracellular Hyp presents clear evidence for Hyp as a compatible solute in *H. halophilus* to cope with osmotic stress.

### Identification of candidate proline 4-hydroxylase-encoding genes in *H. halophilus*

Prior to this study, no gene in the genome of *H. halophilus* in GenBank was annotated as a PH-4, which hydroxylates L-proline to generate Hyp, although we showed here that *H. halophilus* can accumulate Hyp at high NaCl concentrations. It was supposed that Hyp was converted from L-proline because Hyp was not detected in MB (data not shown) and the genome of *H. halophilus* harbors a gene encoding PH-4. PH-4 is a typical protein belonging to the Fe(II)/α-ketoglutarate-dependent hydroxylase family with a conserved α-ketoglutarate- and Fe(II)-dependent oxygenase (2OG-Fe[II] Oxy) domain (Hausinger, 2004; Jia et al., 2017). Thus, we searched for candidate PH-4 genes in the genome of *H. halophilus* based on the presence of a 2OG-Fe[II] Oxy domain-encoding sequence. To search the *H. halophilus* genome, the 2OG-Fe[II] Oxy domain sequences in the PH-4 genes from *Dactylosporangium* sp. (Falcioni et al., 2013), *P. stutzeri* (Yi et al., 2014), and *B. bronchiseptica* RB50 (Yi et al., 2014), which have been demonstrated functionally and experimentally, were used. Three genes containing 2OG-Fe[II] Oxy domain sequences, HBHAL_RS02420, HBHAL_RS11735, and HBHAL_RS15600, which were annotated as a prolyl 4-hydroxylase, multidrug DMT transporter permease, and hypothetical protein, respectively, were retrieved as candidate PH-4 genes.

**Figure 3:**
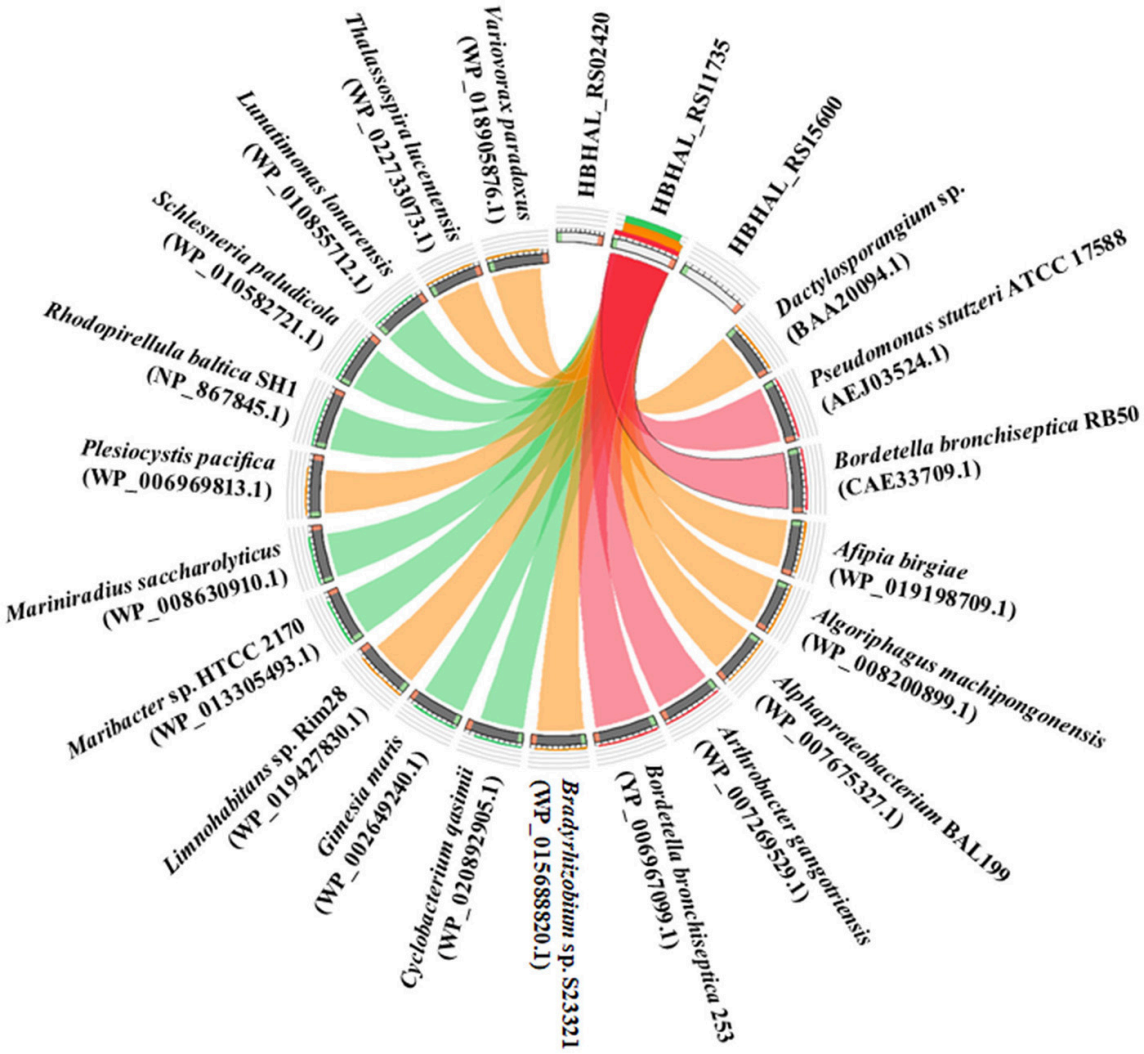
Circos plot showing the relationships between the products of three candidate genes (HBHAL_RS02420, HBHAL_RS11735, HBHAL_RS15600) and proteins annotated as PH-4 in GenBank. The plot was generated with Circoletto. The ribbons between the three candidate proteins and GenBank PH-4 proteins represent their local alignments and the ribbon colors indicate their amino acid sequence identities (no ribbons, <10%; blue, 10–15%; green, 15–25%; orange, 25–40%; red, >40%).

The translated amino acid sequence of HBHAL_RS11735 exhibited 51.0, 49.8, and 29.5% amino acid identities to the PH-4 sequences of *B. bronchiseptica* RB50, *P. stutzeri*, and *Dactylosporangium* sp., respectively, whereas the translated amino acid sequences of HBHAL_RS02420 and HBHAL_RS15600 showed very low sequence identities (<10%) to the reference PH-4 sequences, even though they harbor 2OG-Fe[II] Oxy domain sequences. A relationship analysis of the three candidate PH-4 proteins and protein homologs annotated as PH-4 in GenBank showed that HBHAL_RS11735 was highly related to protein homologs annotated as PH-4 in GenBank, whereas HBHAL_RS02420 and HBHAL_RS15600 showed low relatedness to the PH-4 homologs in GenBank (Figure 3). A phylogenetic tree based on the amino acid sequences of the candidate PH-4 genes also showed that HBHAL_RS11735 was more closely related to the PH-4 proteins of *A. gangotriensis, B. bronchiseptica*, and *P. stutzeri* than HBHAL_RS02420 and HBHAL_RS15600 (Supplementary Figure 2). These results suggest that, of these three candidates, HBHAL_RS11735 is more likely to be the PH-4-coding gene in the genome of *H. halophilus*, even though it was annotated as a multidrug DMT transporter permease.

### Overexpression and activity analysis of candidate PH-4 genes

**Figure 4:**
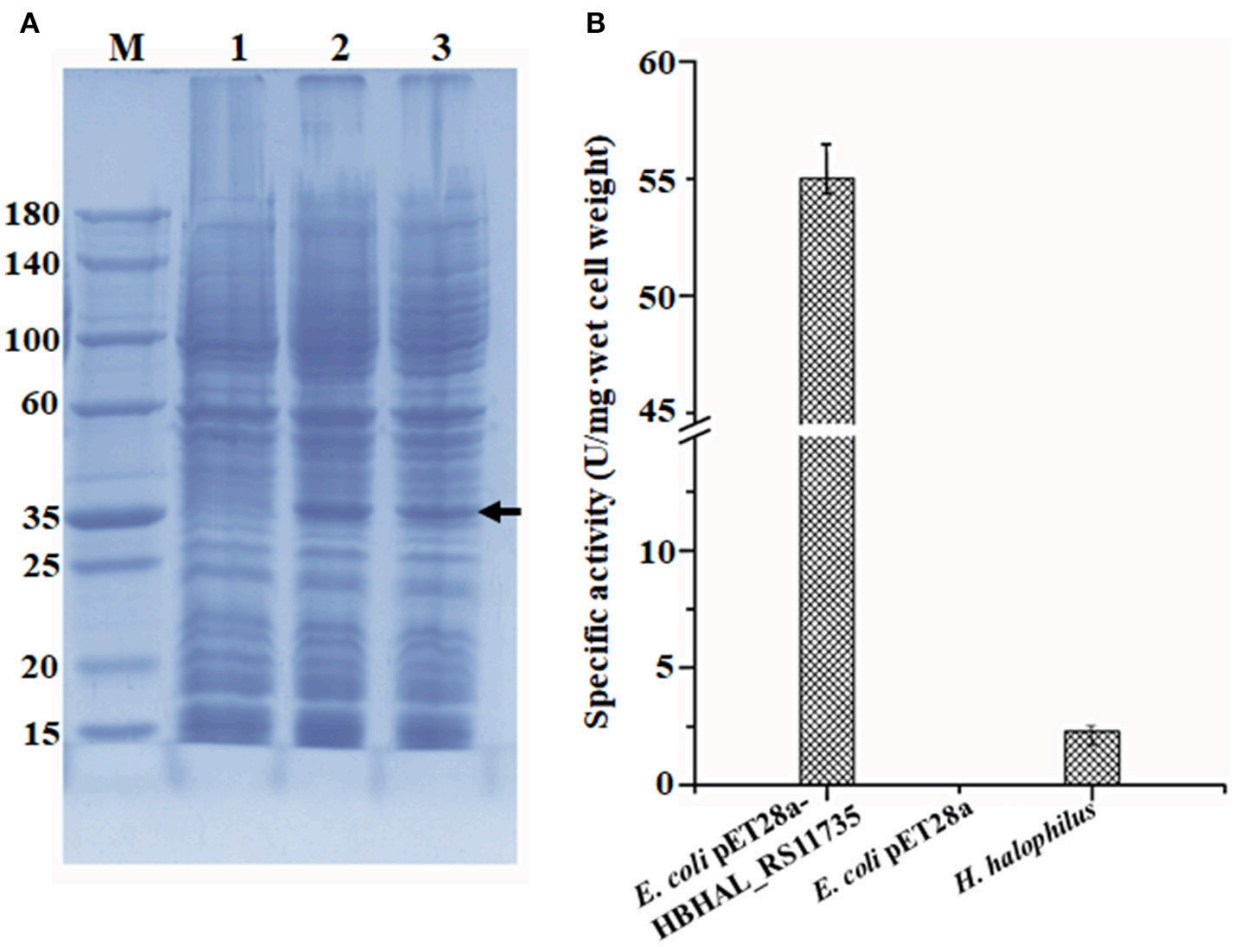
Expressional analysis of HBHAL_RS11735 using a pET28a construct in *E. coli* BL21 (DE3) cells **(A)** and PH-4 activity assay of the overexpressed gene product **(B)**. **(A)** A pET28a-HBHAL_RS11735 construct was overexpressed in *E. coli* BL21 (DE3) cells, and crude protein extracts of these cells were separated by SDS-PAGE. lane M, protein size marker; lane 1, total protein from uninduced cells carrying the HBHAL_RS11735 gene; lane 2, total protein from induced cells carrying the HBHAL_RS11735 gene; lanes 3, soluble protein from induced cells carrying the HBHAL_RS11735 gene. **(B)** PH-4 activity assay of soluble protein from *E. coli* cells overexpressing the HBHAL_RS11735 gene. PH-4 activity is expressed as enzyme units per mg·wet cell weight, and cells of *E. coli* BL21 (DE3) without the HBHAL_RS11735 gene and *H. halophilus* DSM 2266 cells were used as negative and positive controls, respectively.

To confirm the function of HBHAL_RS11735 as a PH-4, catalyzing the hydroxylation of proline to generate Hyp in *H. halophilus*, the gene was cloned under the transcriptional regulation of the IPTG-inducible T7 promoter in pET-28a and transformed into *E. coli* BL21 (DE3) cells. Then, *E. coli* cells harboring pET28a-HBHAL_RS11735 were grown, and the cloned HBHAL_RS11735 was overexpressed. An overexpressed protein band with an approximate molecular mass of 34.2 kDa, the expected size of HBHAL_RS11735, was observed on an SDS-PAGE gel (Figure 4A). The PH-4 activity of a lysate from IPTG-induced *E. coli* BL21 (DE3) cells harboring pET28a-HBHAL_RS11735 was checked by measuring Hyp production from proline. *H. halophilus* and *E. coli* BL21 (DE3) host cells were used as the positive and negative controls, respectively (Figure 4B). *H. halophilus* cell extracts showed weak PH-4 activity (2.3 U/mg·wet cells), while cell extracts of the recombinant *E. coli* BL21 (DE3) cells harboring pET28a-HBHAL_RS11735 showed strong PH-4 activity (55.1 U/mg·wet cell), which was similar to the enzyme activities of *E. coli* cell extracts expressing PH-4-encoding genes derived from *P. stutzeri* (22.2 U/mg·wet cell), *Dactylosporangium* sp. (60.4 U/mg·wet cell), and *B. bronchiseptica* (50.0 U/mg·wet cell) in a previous study (Yi et al., 2014). However, no PH-4 enzyme activity was observed for *E. coli* BL21 (DE3) cells expressing HBHAL_RS02420 or HBHAL_RS15600 (data not shown). In addition, IPTG-induced *E. coli* BL21 (DE3) cells harboring pET28a-HBHAL_RS11735 showed no ectoine hydroxylase activity, i.e., the generation of hydroxyectoine from ectoine (data not shown). These results clearly indicate that HBHAL_RS11735, which was annotated as a multidrug DMT transporter permease in GenBank, encodes the PH-4 that catalyzes the hydroxylation of L-proline to generate Hyp in *H. halophilus*.

### Proline and Hyp biosynthetic gene clusters in *H. halophilus* and their transcriptional expression

**Figure 5:**
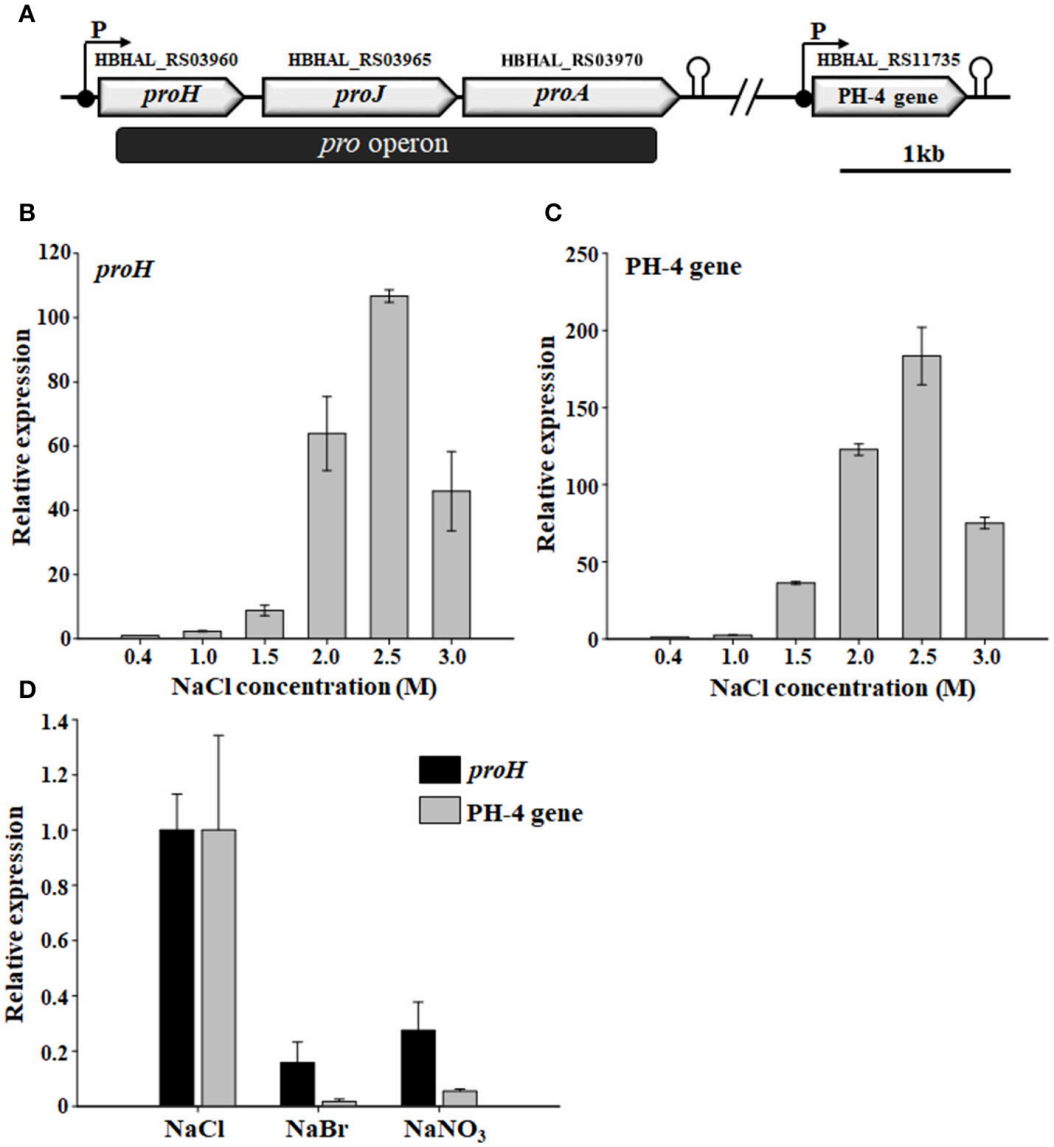
Physical maps of the proline and Hyp biosynthetic gene clusters showing the open reading frames, promoters, and terminators **(A)** and the relative transcriptional expression of *H. halophilus proH* and PH-4 gene in the presence of different NaCl concentrations **(B,C)** and salts **(D)**. The putative functions of HBHAL_RS03960, HBHAL_RS03965, HBHAL_RS03970, and HBHAL_RS11735 were predicted to be pyrroline-5-carboxylate reductase (ProH), glutamate-5-kinase (ProJ), glutamate-5-semialdehyde dehydrogenase (ProA), and proline 4-hydroxylase (PH-4, this study), respectively. Cells grown in marine broth with different NaCl concentrations or salts were used in the transcriptional analysis, and their relative expression in the presence of different NaCl concentrations and salts were calculated based on expressional levels at 0.4 M and 1.0 M NaCl, respectively. The levels of malate dehydrogenase transcript (HBHAL_RS13485, *mdh*) were used for normalization of total RNA templates.

Physical maps of the proline and Hyp biosynthetic gene clusters in *H. halophilus* were proposed based on a BLAST analysis of proline biosynthetic genes, characterization of HBHAL_RS11735, and web-based transcriptional analysis (Figure 5A). As reported previously (Saum and Müller, 2007, 2008a), the proline biosynthetic genes, consisting of *proH* (HBHAL_RS03960), *proJ* (HBHAL_RS03965), and *proA* (HBHAL_RS03970), are arranged as a single transcriptional unit (the *pro* operon), with a single promoter and rho-independent terminator, in the genome of *H. halophilus* (Figure 5A). This arrangement is different from the structure in *B. subtilis*, in which, the proline biosynthetic genes do not constitute a single transcriptional unit (Brill et al., 2011; Kohlstedt et al., 2014). HBHAL_RS11735, encoding a PH-4 that catalyzes the hydroxylation of L-proline at the *trans*-4 position to generate Hyp was distantly located from the *pro* operon in the genome of *H. halophilus*, and the PH-4 gene constituted a single transcriptional unit with a promoter and a rho-independent terminator (Figure 5A).

Because the intracellular levels of proline and Hyp in *H. halophilus* were dependent on the NaCl concentration (Figure 2), an RT-qPCR analysis was conducted to investigate whether the transcriptional expression of the *pro* operon and the PH-4 gene were also dependent on NaCl concentration. The transcriptional expression of *proH* and the PH-4 gene increased markedly as the NaCl concentration increased from 0.4 to 2.5 M NaCl, especially at 1.5–2.5 M NaCl (Figures 5B,C); the transcriptional expression of *proH* and the PH-4 gene were 106 and 183 times higher at 2.5 M NaCl, respectively, than their expression at 0.4 M NaCl. However, the expression was lower at 3.0 M NaCl than at 2.5 M NaCl. These data indicated that the transcriptional expression of *proH* and the PH-4 gene in *H. halophilus* was strongly salinity-dependent, which was in accordance with the intracellular concentrations of proline and Hyp analyzed at various NaCl concentrations (Figure 2), suggesting that proline and Hyp are important compatible solutes in *H. halophilus*.

In addition, to investigate the ability of chloride anion to induce the transcription of the *pro* operon and the PH-4 gene in *H. halophilus*, the expression of *proH* and the PH-4 gene were analyzed in cells grown in MB medium containing different anions (NaCl, NaBr, and NaNO_3_). The results showed that like *proH*, the transcriptional expression of the PH-4 gene was effectively induced by chloride anion when compared to the expression in the presence of other anions (Saum et al., 2006; Figure 5D), which suggests that proline and Hyp may be important compatible solutes for protecting *H. halophilus* from osmotic stress caused by high NaCl concentrations.

### Analysis of intracellular proline and Hyp in other halotolerant and halophilic bacteria

To investigate the use of proline and Hyp as widespread compatible solutes for coping with high salinity, the levels of intracellular proline and Hyp in some halotolerant and halophilic bacteria were analyzed under NaCl optimum and stress conditions. The accumulation of proline and Hyp under high salinity differed depending on the bacteria. *H. halophilus*, other marine bacteria, especially *V. pantothenticus* and *M. stanieri*, clearly accumulated proline and Hyp under high salinity (Table 1), suggesting that Hyp may be an important compatible solute responding to high salinity in *V. pantothenticus* and *M. stanieri. B. subtilis* and *V. siamensis* also accumulated proline and Hyp, although the quantities accumulated were not large. However, *Bordetella bronchiseptica, P. stutzeri, S. halophilum*, and *H. elongata* did not accumulate proline and Hyp under high salinity conditions.

**Table 1:** Intracellular proline and Hyp concentrations in various halotolerant and halophilic bacteria under optimum (Opt) and NaCl stress (Str; corresponding to 1.5–3 times the optimum NaCl concentration) conditions.

	**Strain**	**NaCl (%) in Opt/Str conditions**	**Proline (μmol/g-dry cell)**		**Hyp (μmol/g-dry cell)**	
			**Opt**	**Str**	**Opt**	**Str**
Gram (+)	*Halobacillus halophilus*	10/15	163.4 ± 12.6	248.16 ± 8.8	188.7 ± 2.9	209.5 ± 4.2
	*Bacillus subtilis*	3.5/7	ND	9.8 ± 5.6	0.8 ± 0.2	2.9 ± 0.1
	*Virgibacillus pantothenticus*	6/10	6.5 ± 0.1	1.9 ± 0.2	12.3 ± 1.4	27.2 ± 1.2
	*Virgibacillus siamensis*	10/15	ND	ND	4.1 ± 0.4	2.8 ± 0.6
	*Lentibacillus juripiscarius*	10/15	ND	ND	0.3 ± 0.1	1.1 ± 0.1
	*Lentibacillus salicampi*	6/10	0.7 ± 0.2	ND	1.3 ± 0.70	ND
	*Salimicrobium halophilum*	10/15	ND	ND	ND	ND
Gram (−)	*Maribacter stanieri*	2/5	5.3 ± 0.8	80.0 ± 6.3	4.4 ± 0.8	17.6 ± 1.7
	*Bordetella bronchiseptica*	0.5/1.5	0.8 ± 0.4	ND	0.7 ± 0.2	0.5 ± 0.1
	*Pseudomonas stutzeri*	2/5	ND	ND	0.8 ± 0.1	0.8 ± 0.2
	*Halomonas elongata*	5/15	ND	ND	ND	ND

*ND, not detected*.

## Discussion

A variety of compatible solutes, such as, glutamate, glutamine, glycine betaine, trehalose, proline, ectoine, β-hydroxyectoine, and mannitol, are used to cope with high salinity stress in halophilic and halotolerant bacteria, including *H. halophilus, B. subtilis, A. baylyi*, and *V. parahaemolyticus*, and their compositions vary depending on the bacterium (Hänelt and Müller, 2013; Ongagna-Yhombi and Boyd, 2013; Sand et al., 2013; Zaprasis et al., 2015; Sévin et al., 2016). The accumulation of Hyp in *T. litoralis* and *Brevibacterium* sp. JCM 6894 under high salt conditions was previously reported (Nagata et al., 1996; Lamosa et al., 1998). However, until now, the accumulation of Hyp in response to high osmotic stress has not been reported in other halophilic and halotolerant bacteria, including *H. halophilus*. Moreover, the biosynthesis and regulation of Hyp in response to high salinity had not yet been explored. Although Hyp is not one of the twenty amino acids encoded by genes for protein biosynthesis via translation, it is a major component of animal and plant proteins because it constitutes a large proportion of the collagen in animals and the glycoproteins in the cell walls and root nodules of plants (White et al., 2012). In animals and plants, Hyp is synthesized by hydroxylation of proline residues within specific proteins by prolyl hydroxylases. Generally, bacteria metabolize Hyp released by protein degradation of animals and plants as a carbon and nitrogen source (White et al., 2012). However, bacteria also synthesize and accumulate Hyp via direct hydroxylation of free L-proline by PH-4 (Falcioni et al., 2013; Yi et al., 2014). Although it is known that hydroxyproline is a component of the peptide antibiotics produced by some microorganisms (Katz et al., 1979), clearly, not all of its functions in bacterial cells were known until now, because most bacteria harboring the PH-4 gene do not produce peptide antibiotics containing Hyp (Falcioni et al., 2013; Yi et al., 2014). Here, we suggest that Hyp may function as an important compatible solute in various halophilic and halotolerant bacteria.

Glutamate, glutamine, glycine betaine, proline, and ectoine have been reported as the major compatible solutes in *H. halophilus* cultured in glucose mineral salt (G10) medium (Hänelt and Müller, 2013; Saum et al., 2013). However, the ^1^H-NMR- and HPLC-based analyses of *H. halophilus* cells cultured in marine broth in this study showed that, in addition to these known organics compounds except for ectoine, Hyp, and other unknown organic compounds were accumulated in response to high salinity (Figures 1, 2, and Figure S1). Ectoine, which has been reported as a compound that is accumulated during stationary phase in G10 medium (Saum and Müller, 2008b; Saum et al., 2013), was detected in a very small quantity under high salinity. Our analyses of *H. halophilus* cells cultured in G10 medium also showed that ectoine was relatively highly accumulated in response to high salinity, while Hyp was detected in a very small quantity (data not shown). These results may suggest that the compatible solutes accumulated under osmotic stress in *H. halophilus* differ depending on culture conditions.

Figures 1, 2 clearly showed that *H. halophilus* accumulated a large amount of Hyp in the cytoplasm under high NaCl salinity. However, although the proline biosynthetic gene cluster has been extensively studied (Saum and Müller, 2007; Saum et al., 2013), no gene responsible for the production of Hyp had yet been demonstrated in *H. halophilus*. Our analysis also showed that no gene encoding PH-4, which hydroxylates L-proline to generate Hyp, was annotated in the genome of *H. halophilus* in GenBank. However, this study demonstrated that a gene, HBHAL_RS11735, which was annotated as a multidrug DMT transporter permease-encoding gene, encoded PH-4 through a search for 2OG-Fe[II] Oxy domain-encoding sequences and protein expressional analysis in *E. coli* (Figures 3, 4).

Although hydroxyectoine was not detected in *H. halophilus* cells in previous studies, Saum et al. (2013) reported that the PH-4 gene, HBHAL_RS11735, was a putative ectoine hydroxylase gene (*ectD*) responsible for the hydroxylation of ectoine to generate hydroxyectoine because its deduced amino acid sequence showed high sequence similarities to putative ectoine hydroxylases from other halophilic and halotolerant bacteria (Bursy et al., 2007; Saum et al., 2013). However, the PH-4 gene, HBHAL_RS11735, alone constituted a transcriptional unit and was not clustered with other osmoregulation-related genes in the genome of *H. halophilus*. In addition, because some reports showed that hydroxyectoine was produced in response to elevated temperatures and its production was regulated by the availability of specific electron acceptors in other halophilic bacteria (Aston and Peyton, 2007; Vargas et al., 2008). Therefore, it was reported that HBHAL_RS11735 is unlikely to be involved in osmoregulation in the previous study (Saum et al., 2013). However, our study showed that the overexpressed HBHAL_RS11735 protein had clear proline hydroxylase activity, but not ectoine hydroxylase activity, and the transcriptional expression of HBHAL_RS11735 was critically regulated by osmotic stress, specifically the amount of chloride anion, similar to the proline biosynthetic gene cluster (Saum and Müller, 2007; Figure 5), indicating that that Hyp produced by the HBHAL_RS11735 (PH-4) protein is an important compatible solute responding to osmotic stress caused by NaCl in *H. halophilus*. In addition to *H. halophilus*, other bacteria, including *V. pantothenticus* (a Gram-positive bacterium) and *M. stanieri* (a Gram-negative bacterium), accumulated Hyp under high salinity (Table 1). Previous studies also reported that a thermophilic archaeon, *T. litoralis*, and a halotolerant bacterium, *Brevibacterium* sp. JCM 6894, accumulated Hyp as a compatible solute (Nagata et al., 1996; Lamosa et al., 1998). Therefore, Hyp may be a universal compatible solute that is used by various halophilic and halotolerant bacteria to cope with salt stress. It is known that high salt conditions can cause oxidative stress in cells, and compatible solutes function as antioxidants to reduce the resulting cell damage (Cuin and Shabala, 2008). It was reported that Hyp has antioxidant activity against oxidative stress, similar to hydroxyectoine, which has a similar chemical structure (Triantis et al., 2007; Argandoña et al., 2010), suggesting that Hyp may function as an osmoprotectant as well as an antioxidant to protect cells under high osmotic conditions.

In conclusion, we demonstrated that *H. halophilus* accumulates Hyp as a major compatible solute in response to high salinity (NaCl), and we identified and characterized the NaCl-induced PH-4 protein that is essential for the hydroxylation of L-proline into Hyp. However, although the transcription of the proline biosynthetic genes and the PH-4 gene are clearly regulated by NaCl, no regulators were identified near the gene clusters. Therefore, further studies are needed to investigate the regulatory mechanisms of these gene clusters in response to osmotic NaCl stress.

## Author contributions

CJ conceived the ideas and supervised the work. KK developed the concepts and KK and BJ performed the experiments and analyzed the data. KK and CJ wrote the manuscript. The manuscript has been reviewed and edited by all authors.

### Conflict of interest statement

The authors declare that the research was conducted in the absence of any commercial or financial relationships that could be construed as a potential conflict of interest.
