# Supplementary material for: Identification of Trans-4-Hydroxy-L-Proline as a Compatible Solute and Its Biosynthesis and Molecular Characterization in Halobacillus halophilus
Source: Front Microbiol. 2017 Oct 20;8:2054. doi: 10.3389/fmicb.2017.02054 (PMC5655121; doi:10.3389/fmicb.2017.02054)
Supplement: Supplementary file 1 [file Presentation1.PDF]

Supplementary Material

Identification of trans-4-hydroxy-L-proline as a compatible solute and its biosynthesis and molecular characterization in *Halobacillus halophilus*

Kyung Hyun Kim†, Baolei Jia† and Che Ok Jeon*

Department of Life Science, Chung-Ang University, Seoul 06974, Republic of Korea

*Corresponding author: Che Ok Jeon, Ph.D.

†These authors contributed equally to this study.

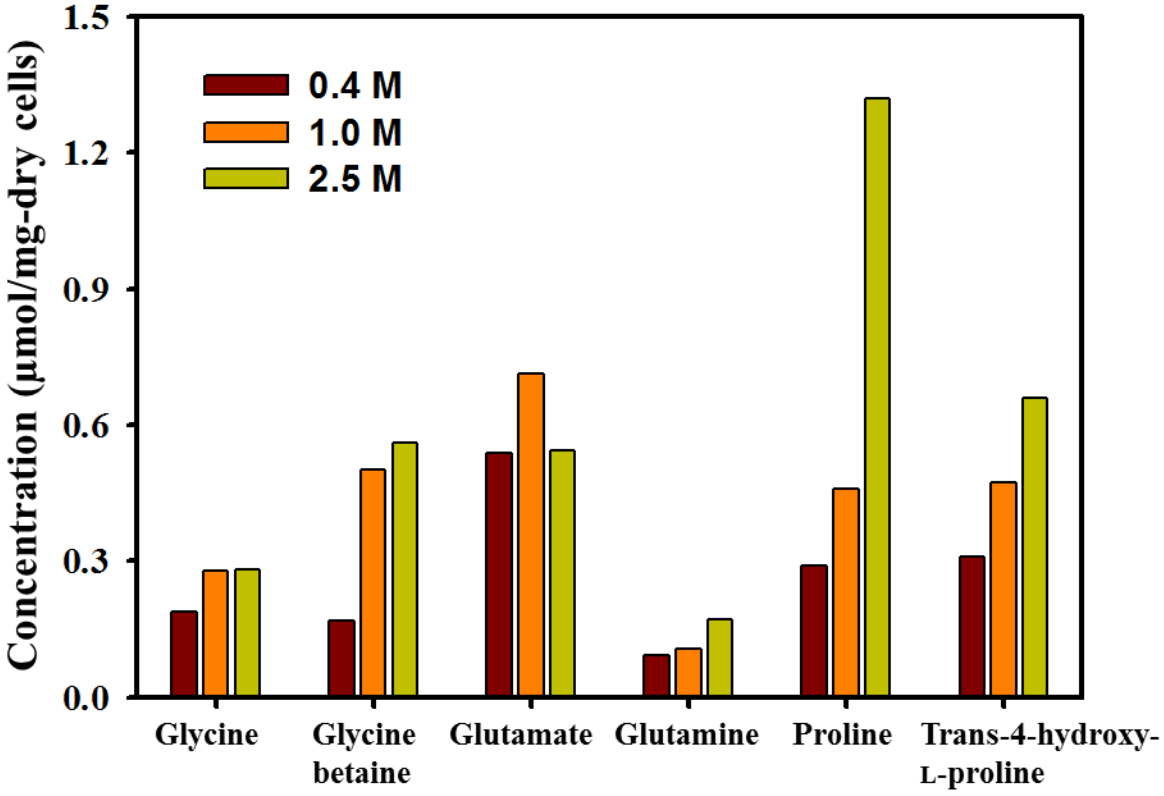

Supplementary Figure S1. Concentrations of the intracellular organic compounds detected in *H. halophilus* grown in marine broth with different NaCl concentrations. The intracellular organic compounds were analyzed with ^{1}H-NMR spectroscopy, and ^{1}H-NMR peaks were identified and quantified with the Chenomx NMR suite (ver. 6.1) using 2,2-dimethyl-2-silapentane-5-sulfonate (DSS) as the internal standard.

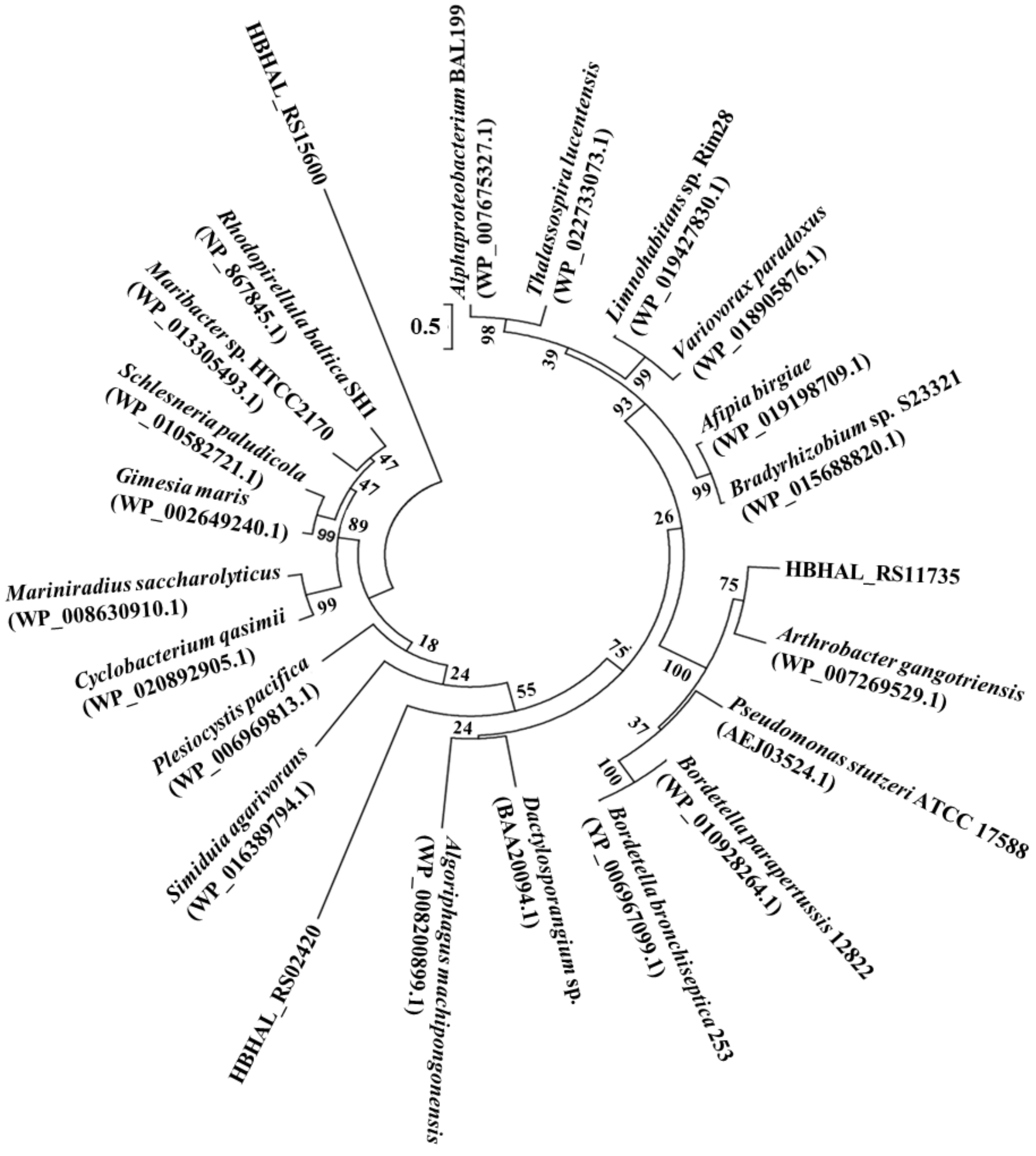

Supplementary Figure S2. A phylogenetic tree based on the maximum likelihood algorithm showing the relationships among the three PH-4 candidate proteins (HBHAL_RS02420, HBHAL_RS11735, HBHAL_RS15600) and reference proteins annotated as PH-4 in GenBank. Numbers on branches indicate bootstrap values expressed as percentages of 1000 replicates. The scale bar represents 0.5 changes per amino acid positions.

Supplementary Table S1. Primers used in this study

Target (putative function) *	Primer	Sequence (5′–3′)†	Product size
For protein overexpression			
HBHAL_RS02420 (prolyl 4-hydroxylase)	prolyl-1F<br>prolyl-607R	C<u>GAATTC</u>ATGACAATGCCAGTGAAAG (EcoRI)<br>CCC<u>AAGCTT</u>TTACCTGTATTGTTTTCTTCTTAC (HindIII)	311 aa
HBHAL_RS11735 (multidrug DMT transporter permease)	per-5F<br>per-872R	CATG<u>CCATGG</u>AAGATCTTTATCCATC (NcoI)<br>CCG<u>CTCGAG</u>TTATTTGTTTACTTTTAGTTTAG (XhoI)	297 aa
HBHAL_RS15600 (hypothetical protein)	hyp-1F<br>hyp-916R	CATG<u>CCATGG</u>CTACTTTACAAAATGTCCT (NcoI)<br>CCG<u>CTCGAG</u>GAAAGAAGTAAAGATGACGTGTGTA (XhoI)	311 aa
For RT-qPCR			
HBHAL_RS03960 (pyrroline-5-carboxylate reductase)	proH-78F<br>proH-198R	GAACATTTCAGCAGATCAG<br>GCCAGAATAAGTTGATCC	137 bp
HBHAL_RS11735 (multidrug DMT transporter permease)	per-221F<br>per-399R	ATCAGGACAGTGAATCGGATATG<br>CCG GTA AAT CCA GGC TTG TAA	199 bp
HBHAL_RS13485 (malate dehydrogenase)	mdh-551F<br>mdh-663R	TGGTCCCATTAACTCGCTATTC<br>CGTTTCCAAGCAGACCTACA	132 bp

† Restriction enzyme sites for cloning are underlined. F (forward) and R (reverse) are shown to indicate the primer direction.

* Locus tags and putative functions were derived from the genome of *H. halophilus* DSM 2266 in GenBank.
